# The impact of sleep disordered breathing on cardiac troponin in acutely decompensated heart failure

**DOI:** 10.1007/s11325-022-02646-7

**Published:** 2022-05-31

**Authors:** Matthew P. Light, Kimberly Y. Kreitinger, Euyhyun Lee, Pamela N. DeYoung, Avni Lakhani, Brent Siegel, Lori B. Daniels, Atul Malhotra, Robert L. Owens

**Affiliations:** 1 Division of Pulmonary, Critical Care and Sleep Medicine, Department of Medicine, University of California San Diego (UCSD), 9300 Campus Point Drive #7381, La Jolla, CA 92037 USA; 2 Altman Clinical and Translational Research Institute, University of California San Diego, La Jolla, CA USA; 3 Division of Cardiology, Department of Medicine, University of California San Diego (UCSD), La Jolla, CA USA

**Keywords:** Sleep apnea, Heart failure, Troponin, Cheyne-Stokes

## Abstract

**Purpose:**

Sleep disordered breathing in decompensated heart failure has physiological consequences (e.g., intermittent hypoxemia) that may predispose to subclinical myocardial injury, yet a temporal relationship between sleep apnea and troponin elevation has not been established.

**Methods:**

We assessed the feasibility of performing respiratory polygraphy and measuring overnight high-sensitivity cardiac troponin T change in adults admitted to the hospital with acutely decompensated heart failure. Repeat sleep apnea tests (SATs) were performed to determine response to optimal medical heart failure therapy. Multivariable logistic regression was used to identify associations between absolute overnight troponin change and sleep apnea characteristics.

**Results:**

Among the 19 subjects with acutely decompensated heart failure, 92% of SATs demonstrated sleep disordered breathing (apnea–hypopnea index [AHI] > 5 events/h). For those with repeat SATs, AHI increased in 67% despite medical management of heart failure. Overnight troponin increase was associated with moderate to severe sleep apnea (vs. no to mild sleep apnea, odds ratio (OR = 18.4 [1.51–224.18]), central apnea index (OR = 1.11 [1.01–1.22]), and predominantly central sleep apnea (vs. obstructive, OR = 22.9 [1.29–406.32]).

**Conclusions:**

Sleep apnea severity and a central apnea pattern may be associated with myocardial injury. Respiratory polygraphy with serial biomarker assessment is feasible in this population, and combining this approach with interventions (e.g., positive airway pressure) may help establish if a link exists between sleep apnea and subclinical myocardial injury.

## Introduction

Heart failure affects 6 million adults in the USA who are prone to periodic decompensation, often requiring hospitalization [1]. This complex syndrome has many triggers and clinical/hemodynamic profiles but is invariably characterized by elevated left ventricular end-diastolic pressure (LVEDP), increased extracellular fluid volume, and symptoms of dyspnea [2]. Another long recognized sign of heart failure decompensation has been central apnea in a Cheyne-Stokes pattern, thought to occur because of increased chemosensitivity in the setting of rising LVEDP and increased circulatory time (among other factors). More recently, it has been recognized that obstructive sleep apnea (OSA) is very common in patients with congestive heart failure, particularly those with heart failure with preserved ejection fraction [3].

The severity of both obstructive and central sleep apnea as measured by the apnea–hypopnea index (AHI) is exaggerated during acutely decompensated heart failure (ADHF) [4, 5] through multiple putative mechanisms, some which are controversial, including rostral fluid shifts with edema of the upper airway [6–9]. Whether obstructive or central apnea, there are acute physiological changes that may be deleterious to the heart, including intermittent hypoxemia (leading to oxidative stress), arousal from sleep with sympathoexcitation [10], and changes in intrathoracic pressure that increase afterload [11–14] and can impair coronary blood flow [15, 16]. Data suggest a benefit to early diagnosis and treatment of moderate to severe sleep disordered breathing (SDB) in ADHF. Khayat et al. have shown improvement in left ventricular ejection fraction (LVEF) within days after initiating therapy for OSA [17]. Similarly, in the CATHF trial, subjects with preserved LVEF randomized to adaptive servo-ventilation had a trend towards reduction in the composite outcome (including death, cardiovascular hospitalization, and change in 6-min walk distance) at 6 months compared with optimal medical therapy alone, although these findings have not been consistent in other studies. [18]

Based on these findings, many have hypothesized that SDB may lead directly to cardiac injury. Cardiac troponin is a sensitive marker of myocardial injury. Roca et al. demonstrated a sex-specific relationship been sleep apnea and troponin elevation, while other studies have been largely inconclusive and have not focused on patients with ADHF who should be considered at risk [19–22]. By combining serial troponin measurements (pre- and post-sleep) using a highly sensitive assay with portable respiratory polygraphy deployed in the inpatient setting, this study was conducted to test the hypothesis that sleep apnea is associated with overnight subclinical myocardial injury in patients with ADHF.

## Methods

### Enrollment

In this prospective observational study, patients 18–80 years of age and admitted to University of California San Diego Health with a primary diagnosis of ADHF were asked to participate and provide written informed consent. Enrolled subjects then underwent two portable sleep apnea tests (SATs) on hospital nights 1 or 2, and again prior to discharge. Evening and morning serum samples were collected for highly sensitive cardiac troponin T (hs-cTnT) analysis. To date, few have attempted portable SATs on inpatients with decompensated heart failure, and little is known about the degree of overnight troponin change in this population. As such this research was conducted primarily to determine the feasibility of this approach. The University of California, San Diego Human Research Protections Program approved this study. This study was registered on clinicaltrials.gov (NCT03804827).

### Exclusion criteria

Exclusion criteria were as follows: (1) acute coronary syndrome suspected by the inpatient treatment team; (2) continuation of outpatient treatment for sleep disordered breathing during hospitalization (e.g., CPAP use during inpatient stay); (3) respiratory failure requiring non-invasive ventilation; (4) patients in intensive care or those requiring advanced heart failure therapy (e.g., inotrope infusion, use of a mechanical circulatory assist device); (5) inability to tolerate study procedures (e.g., respiratory polygraphy); (6) attending physician refusal.

## Initial assessment

Demographic and baseline clinical data were collected from the electronic medical record and included age, gender, body mass index (BMI), vital signs including oxyhemoglobin saturation (and need for supplemental oxygen), comorbid conditions, medications, and laboratory values and studies if available (e.g., troponin, N-terminal pro B-type natriuretic peptide, creatinine, chest x-ray, echocardiogram). In addition, subjects were asked to complete the Epworth Sleepiness Scale to measure baseline daytime sleepiness, and the Berlin questionnaire which stratifies patients into low- or high-risk of obstructive sleep apnea. All data were stored securely in REDCap (Research Electronic Data Capture, REDCap Consortium).

## Sleep apnea testing

Subjects underwent an initial sleep apnea test on either the evening of hospital admission or the evening of hospital day 1. The portable sleep apnea testing device (ApneaLink, Resmed, San Diego, CA) was applied at bedtime and measures heart rate, oxyhemoglobin saturation, nasal airflow, and respiratory effort. In the morning, the device was removed after 6–8 h of recording time. Each overnight recording was then manually scored by a certified sleep technician blinded to any clinical information. For those subjects being treated with supplemental oxygen at the time of respiratory polygraphy, the following medical center protocol was applied: The respiratory therapist evaluated the need for oxygen prior to the sleep test by obtaining a room air SpO2 (patients on supplemental oxygen had it removed 15 min prior to obtaining a SpO2). Supplemental oxygen was only administered during the study if saturations were < 88%. In the event the patient required supplemental oxygen, a Venti mask was used over the nasal airflow sensor and the FiO2 was set to achieve an SpO2 > 88%. During the sleep study, if resting SpO2 fell below 88%, the FiO2 was increased.

### Assessing for overnight, subclinical myocardial injury

In association with each SAT, phlebotomy was performed pre- (06:00–10:00 PM) and post-sleep (04:00–08:00 AM). Samples were immediately processed into serum and stored at − 80 °C. Samples were then analyzed in batch for levels of hs-cTnT (Meso Scale Diagnostics, Rockville MD).

## Follow-up

For each hospital day, clinical data were recorded including vital signs, serum chemistries, and interval urine output and weight if available. To determine if a period of medical heart failure management affected SDB-associated troponin release, subjects were offered a second SAT (again with pre- and post-troponin measurements) following the same procedure as the initial phase of testing.

## Statistical analysis

All data were tested for normality using the Shapiro–Wilk method and described either with means and standard deviations (normal distribution) or non-parametric statistics when appropriate. SATs were scored according to American Academy of Sleep Medicine rules as follows: apnea was defined as absence of airflow (< 10% baseline) for > 10 s, and hypopnea was defined as 30% reduction in airflow for > 10 s resulting in at least 3% oxygen desaturation (AHI3%) [23]. The apnea–hypopnea index was calculated in standard fashion. For all apneas, the presence or absence of respiratory effort was used to distinguish obstructive from central events respectively. Sleep apnea severity was determined by the AHI as follows: AHI 0–15/h: no-mild sleep apnea; AHI ≥ 15/h moderate-severe sleep apnea. The presence or absence of Cheyne-Stokes was assessed using the AASM Rules for Scoring Respiratory Evens in Sleep; i.e., there are episodes of ≥ 3 consecutive central apneas and/or central hypopneas separated by a crescendo and decrescendo change in breathing amplitude with a cycle length of at least 40 s (typically 45 to 90 s). Once the presence of CSA-CSR was confirmed by the RPSGT, the percentage of time in CSR was quantified by the manufacturer scoring algorithm. SATs in which the central apnea index (CAI: central apneas per hour of valid signal time) was greater than 0.5 X AHI (i.e., there were more central apneas than the sum of obstructive apneas and hypopneas) were further classified as predominantly central sleep apnea, otherwise SATs were classified as predominantly obstructive. For each SAT, T90 (percentage of valid signal time with SaO_2_ < 90%), T80 (percentage of valid signal time with SaO_2_ < 80%), and oxygen desaturation index (ODI, desaturation events/h of valid signal time) were calculated.

Overnight change in cardiac troponin was calculated by subtracting the evening (pre-sleep) value from the morning (post-sleep) value and was categorized into either overnight increase or overnight decrease. Each night of valid respiratory polygraphy with an associated overnight troponin was then aggregated to create a final data set, noting repeated measures for some subjects. To determine associations between SDB characteristics and overnight troponin change (binary outcome, increase or decrease), we fitted univariate generalized mixed effects models with a binomial distribution and random intercept. Statistical analyses were performed using Stata (version 16.1, StataCorp LLC, College Station, TX) and R (R Core Team 2020, R Foundation for Statistical Computing, Vienna, Austria). The dataset generated and analyzed during the current study are available from the corresponding author on reasonable request.

## Results

Between January and December 2019, 23 subjects were recruited and consented to participate in the study. A total of 19 subjects (83%) completed an initial SAT; of those, 12/19 (63%) underwent a follow-up SAT prior to hospital discharge. A total of 26 nights of respiratory polygraphy with associated troponin levels were collected. For those subjects who did not complete initial sleep apnea testing, the primary reason was device intolerance in 3/4 (75%).

Subject demographics and baseline (admission) clinical data are provided in Table 1. Nine subjects (39%) were female, and the average age was 67 years (range 49–78 years). All subjects had a pre-existing diagnosis of heart failure (i.e., no subjects had new-onset heart failure), and most subjects fit a clinical hemodynamic profile of mild to moderate ADHF (78%). Hypertension (84%) and diabetes (47%) were common comorbidities among subjects, and 53% had pre-existing CKD. Sixteen percent had a prior myocardial infarction and 16% had a history coronary artery bypass grafting; 11% had prior percutaneous coronary intervention. Most subjects (63%) had a reduced LVEF. High-sensitivity troponin T was detectable on admission for all subjects who had the test ordered as part of their clinical care. The mean Epworth Sleepiness Scale was 10 (range 2–21), and 74% of subjects were assessed to be high-risk of obstructive sleep apnea based on the Berlin score.

**Table 1:** Characteristics of 19 subjects hospitalized with acutely decompensated heart failure who completed respiratory polygraphy with associated pre- and post-sleep troponin analysis, stratified by sleep apnea severity on initial SAT (no-mild vs. moderate-severe)

	All subjects	No-mild sleep apnea.^a^	Mod-severe sleep apnea.^b^
Characteristic	(*n* = 19)	(*n* = 9)	(*n* = 10)
Age, mean (SD)	67.4 (7.8)	69.1 (5.8)	65.9 (9.3)
Sex, *n* (% male)	13 (68)	5 (56)	8 (80)
BMI (kg/m.^2^), mean (SD)	30.2 (6.9)	30.7 (7.9)	29.7 (6.3)
Hypertension, *n* (%)	16 (84)	7 (78)	9 (90)
Diabetes, *n* (%)	9 (47)	3 (33)	6 (60)
Atrial fibrillation, *n* (%)	9 (75)	5 (56)	4 (40)
CKD, *n* (%)	10 (53)	4 (44)	6 (60)
Prior MI, *n* (%)	3 (16)	1 (11)	2 (20)
Prior CABG, *n* (%)	3 (16)	0 (0)	3 (30)
Prior PCI.^c^, *n* (%)	2 (11)	1 (11)	1 (10)
LVEF, mean (SD)	38 (16)	39 (19)	37 (14)
LVEF classification.^d^, *n* (%)			
Preserved	5 (26)	2 (22)	3 (30)
Mid-range	2 (11)	2 (22)	0 (0)
Reduced	12 (63)	5 (56)	7 (70)
Admission labs			
Troponin T (ng/L), mean (SD)	37 (22)	27 (7)	44 (27)
NT-proBNP (pg/mL), median (IQR)	2903 (1362)	2903 (1189)	2910 (3492)
Sodium (mmol/L), mean (SD)	141 (4)	141 (3)	140 (5)
Creatinine (mg/dL), median (IQR)	1.3 (0.8)	1.2 (0.6)	1.3 (0.5)
Epworth Sleepiness Scale, mean (SD)	10 (5)	7 (5)	13 (5)

*BMI* body mass index, *NT-proBNP* N-terminal fragment of pro B-type natriuretic peptide, *CABG* coronary artery bypass graft, *CKD* chronic kidney disease, *LVEF* left ventricular ejection fraction, *MI* myocardial infarction.

^a^No-mild sleep apnea: AHI < 15 on initial SAT

^b^Moderate-severe sleep apnea: AHI ≥ 15 on initial SAT

^c^Subjects with prior percutaneous coronary intervention (stent or balloon angioplasty)

^d^Preserved: LVEF ≥ 50%; mid-range: LVEF 40–49%; reduced: LVEF < 40%

SAT results for 26 nights of respiratory polygraphy are shown in Table 2. Regarding initial sleep apnea testing (night 1, *n* = 19), the median AHI was 18 events/h (range 1–59 events/h). A total of 32% of SATs were consistent with mild sleep apnea, while 16% and 37% were consistent with moderate and severe sleep apnea, respectively. The median AHI for all nights of monitoring (*n* = 26) was 22 events/h (range 1–67 events/h). An AHI > 5 events/h was observed on 92% of SATs completed; 30% were classified as mild sleep apnea, 27% were moderate, and 35% were severe. Oxyhemoglobin desaturations were common as reflected by both the T90 (median 34% of valid signal time) and the ODI (mean 25 events/h). Cheyne-Stokes respirations were observed in 46% of SATs, although a majority of studies were classified as primarily obstructive sleep apnea (69%).

**Table 2:** Results from 26 nights of respiratory polygraphy in hospitalized patients with acutely decompensated heart failure and stratified by overnight troponin T change (increase vs. decrease)

	All nights	Troponin T increase.^a^	Troponin T decrease.^a^
Test result	(*n* = 26)	(*n* = 11)	(*n* = 15)
AHI.^b^ (events/h), median (IQR)	21.6 (35.3)	44.6 (36.3)	14.9 (19.6)
CAI.^c^ (events/h), median (IQR)	3.0 (22.5)	22.7 (36.2)	0.6 (9.0)
T90.^d^ (%), median (IQR)	34.0 (51.0)	44.0 (36.0)	23.0 (83.0)
ODI.^e^ (events/h), mean (SD)	24.8 (18.0)	30.9 (18.4)	20.4 (17.0)
CSR present, *n* (%)	12 (46)	5 (45)	7 (47)
SDB type.^f^, *n* (%)			
Central	8 (31)	6 (55)	2(13)
Obstructive	18 (69)	5 (45)	13(87)

*AHI* apnea–hypopnea index, *CAI* central apnea index, *CSR* Cheyne-Stokes respirations, *ODI* oxygen desaturation index, *SDB* sleep disordered breathing.

^a^Refers to overnight troponin change (increase vs. decrease)

^b^AHI4% (4% desaturation is required for events to be classified as apnea)

^c^Central apneas per hour of valid signal time during respiratory polygraphy

^d^Percentage of valid signal time with an oxygen saturation < 90%

^e^Desaturation events ≥ 4% per hour of valid signal time

^f^Predominant respiratory event type (central vs. obstructive) determined by comparing the AHI and CAI (methods)

For those subjects who agreed to serial SATs during the course of the study (*n* = 12), the median AHI of the first and second SATs respectively were 13 (IQR 5–43) and 23 (IQR 12–48). Overall, 8/12 subjects had an increase in their AHI on repeat testing. In 3/12 subjects, severity increased from no-mild sleep apnea into the moderate-severe range, while no subjects improved from moderate-severe to no-mild disease. In only one subject did the predominant SDB type change (from obstructive to central sleep apnea) on serial assessment. It should be noted that during hospitalization, the average weight lost was 6.0 ± 4.3 kg, presumably due to diuresis. All subjects lost weight during admission.

**Fig. 1:**
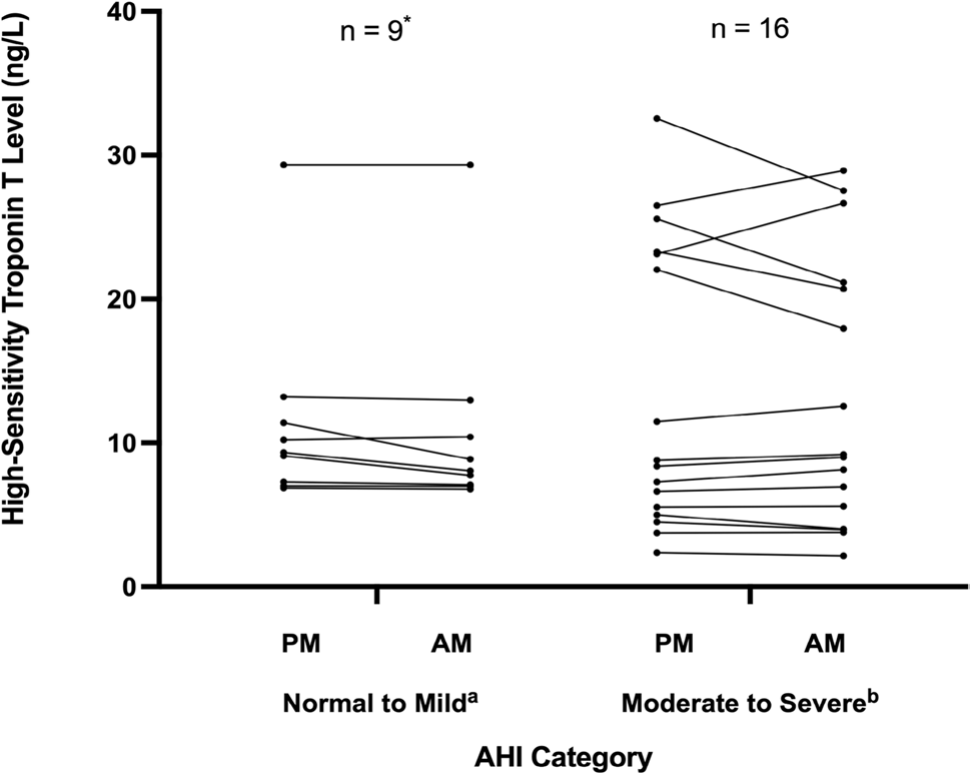
Pre- (PM) and post-sleep (AM) high-sensitivity troponin T levels in subjects with no-mild vs. moderate-severe sleep apnea. Abbreviations: AHI, apnea–hypopnea index: (**a**) no-mild sleep apnea by AHI (AHI < 15 events h); (**b**) moderate-severe sleep apnea by AHI (AHI ≥ 15 events/h). ^*^One outlier with severely elevated troponin T levels (pre-sleep: 56.40 ng/L, post-sleep: 65.11 ng/L) was excluded from the normal-mild sleep apnea group to optimize the y-axis scale

Mean pre-sleep hs-cTnT level was 15 ng/L (range 2–56) compared with a mean post-sleep hs-cTnT level of 14 ng/L (range 2–65). Hs-cTnT overnight percent change ranged from − 22 to + 15%. In total, there were 15 nights in which hs-cTnT decreased (58%) and 11 nights in which troponin increased (42%). Pre- and post-sleep hs-cTnT levels for all nights of monitoring are shown in Fig. 1. For those 12 subjects who underwent multiple assessments, 4 subjects (33%) had a troponin increase on both nights, and 2 subjects (17%) had an initial troponin decrease followed by an overnight increase.

**Fig. 2:**
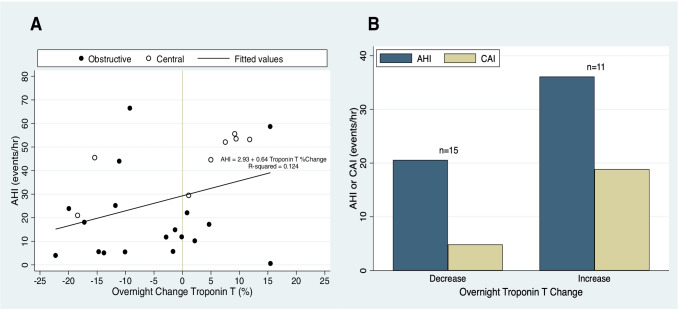
**A** Overnight percent change in high-sensitivity cardiac troponin T (hs-cTnT) vs. apnea–hypopnea index (AHI, events/h) during 26 nights of monitoring in subjects admitted to the hospital with acutely decompensated heart failure. The predominant sleep apnea phenotype (central vs. obstructive) is indicated. **B** Mean AHI and central apnea index (CAI) tended to be higher during nights with an overnight increase in hs-cTnT (*n* = 11) vs. those with an overnight decrease (*n* = 15). Abbreviations: AHI, apnea–hypopnea index; CAI, central apnea index

Combining results from all nights of respiratory polygraphy, logistic mixed model regression indicated that overnight hs-cTnT increase was associated with moderate-severe sleep apnea (vs. no-mild, OR = 18.4 [1.5–224.2]), central apnea index (CAI, OR = 1.1 [1.0–1.2]), and predominantly central sleep apnea (vs. predominantly obstructive, OR = 22.9 [1.3–406.3]). Figure 2 shows the overnight percent change in hs-cTnT in relation to the AHI (Panel A), and compares the AHI and CAI of subjects with overnight troponin increase vs. decrease (Panel B). The apnea–hypopnea index (AHI), apnea index (AI), T90, T80, ODI, and presence of Cheyne-Stokes breathing were not associated with overnight hs-cTnT change.

## Discussion

In this observational pilot study of 19 subjects with ADHF who underwent a total of 26 nights of monitoring, 92% of SATs were abnormal, and 62% were consistent with moderate-severe sleep apnea. OSA was more common than central sleep apnea though Cheyne-Stokes respirations were observed in nearly half of those with a predominantly obstructive phenotype, indicating mixed disease. In addition, on serial assessment during the course of hospitalization, an increase in the AHI was observed in 67% of SAT pairs. Finally, hs-cTnT levels increased during the night in approximately 40% of observations, and moderate-severe sleep apnea (AHI ≥ 15/h), a predominantly central sleep apnea phenotype (vs. obstructive) and central apnea index predicted an overnight increase in hs-cTnT concentration. SATs were generally well-tolerated in this patient population (83% completed an initial SAT) and provided enough valid signal time for adequate SDB assessment.

With respect to the prevalence of sleep disordered breathing, our findings are similar to others’ who have previously observed a high proportion of subjects with sleep apnea (as defined by AHI ≥ 5 events/h) in the setting of ADHF (roughly 90%). In addition, our cohort had a similar proportion of OSA (vs. central) as the study by Aurora et al., but differed from the predominantly Cheyne-Stokes breathing observed by Padeletti et al. who limited their evaluation to patients with an LVEF < 40% [4, 5]. It should be noted that portable sleep monitoring, while accurate, tends to underestimate sleep apnea severity (compared with polysomnography) in this population [4], which we speculate may be partially related to supplemental oxygen therapy which can mask hypopneas. SATs might be less reliable for the assessment of central vs. obstructive events, however, prior literature actually suggests very good agreement comparing PSG to the SAT equipment we used [4]. Moreover, portable monitoring frequently relies on assuming the patient is asleep rather than measuring it, leading to a further reduction in the AHI particularly if sleep quality is poor [24]. Thus, our findings likely underestimate the true burden of sleep apnea.

We were surprised to find that sleep apnea severity (as measured by the AHI) tended to increase throughout the course of hospitalization in subjects with multiple nights of monitoring, despite inpatient treatment for ADHF. Few prior studies have attempted multiple sequential inpatient sleep apnea assessments in this population; however, Padeletti et al. found that time in Cheyne-Stokes breathing did not improve, and nadir S_a_O_2_ actually increased on short-interval follow-up [5]. In summary, sleep apnea (both obstructive and central) was common, and treatment for ADHF alone was insufficient to acutely resolve most observed sleep disordered breathing.

Rising troponin has been observed previously in patients with ADHF. In an analysis from the PROTECT pilot study in which cTnT (contemporary and not high sensitivity) was measured daily, O’Connor et al. found 21% of subjects converted from undetectable to a detectable troponin level [25]. A smaller study by Del Carlo et al. showed 11% of subjects developed a detectable troponin T (also not high sensitivity) after 7 days. [26] In contrast, we observed an increase in hs-cTnT overnight during approximately 40% of nights monitored. While these results (at least in part) are likely due to increased assay sensitivity, it raises the possibility of ongoing, subclinical myocardial injury despite initiation of treatment for ADHF. If confirmed, this finding would suggest that early recognition and treatment of sleep disordered breathing may present an opportunity to improve outcomes in ADHF.

The exact mechanisms of troponin elevation in AHDF are unknown. Many postulate sub-endocardial ischemia along with other processes such as increased filling pressures/wall stress, endothelial dysfunction, and tachycardia or arrhythmia [27]. Clearly, sleep apnea can produce many of these undesired cardiovascular consequences and as such a causal link between sleep disordered breathing and troponin elevation in ADHF is plausible. If true, however, it is important to determine which aspect of sleep apnea (e.g., number/type of event, burden of hypoxemia) is most pathogenic in order to guide further research and therapy. Several studies have now documented an association between elevated AHI and increased high-sensitivity troponin levels, most recently Raut et al. (2021) [28], and in this regard our regression finding that an AHI in the moderate-severe range predicts troponin T elevation (vs. AHI < 15/h) in patients with ADHF is consistent. Finally, while both a predominantly central sleep apnea phenotype on SAT (vs. obstructive), and central apnea index appeared to predict overnight hs-cTnT increase, whether this association reflects the sleep disorders independent of underlying heart failure severity in unknown and worthy of future study.

A key strength to this study was the design in which both pre- and post-sleep serum samples were collected such that overnight change in hs-cTnT (as opposed to morning/daily levels) could be determined in relation to each SAT. An important weakness was the small sample size. Serum hs-cTnT levels are influenced by a variety of physiologic processes and the limited size of this study precludes adjustment for all potential confounders (e.g., creatinine clearance) [20]. In addition, the absence of electroencephalographic data during portable monitoring tends to underestimate sleep apnea severity in those with fragmented or inefficient sleep (e.g., insomnia) and may have influenced our results [24]. In this regard, first-night SATs may have been disproportionately affected by disruptions related to clinical care. Another important limitation related to technique was difficulty classifying hypopneas as either central or obstructive. With measurement of hypopneas as central or obstructive, there may be some subjects who have central sleep apnea but may have been misclassified by us as obstructive sleep apnea. However, this does not change our finding that the central sleep apnea phenotype — defined based on apneas — is associated with overnight troponin increase. Lastly, women were relatively underrepresented in our sample, although reflective of our inpatient population. [22]

## Conclusions

Most patients with ADHF have sleep disordered breathing while hospitalized, which in the acute setting responds poorly to conventional heart failure therapy alone. In addition, overnight troponin increase is common (40%) and may persist throughout hospitalization. Both sleep apnea severity and a predominantly central sleep apnea phenotype are associated with overnight hs-cTnT increase. Interventional studies with pre- and post-sleep biomarker assessment are needed to determine if a clear association between sleep apnea and subclinical myocardial injury exists, and whether or not early in-hospital assessment and treatment of sleep apnea has the potential to shift the paradigm of decompensated heart failure care.

## Data Availability

The dataset generated and analyzed during the current study are available from the corresponding author on reasonable request.
